# Preparation of the heterojunction catalyst N-doping carbon quantum dots/P25 and its visible light photocatalytic activity

**DOI:** 10.1038/s41598-019-46277-7

**Published:** 2019-07-10

**Authors:** Ning Xu, Hongqin Huang, Hao Ouyang, Huigang Wang

**Affiliations:** Department of Chemistry, Zhejiang Sci-Tech University, Hangzhou, 310018 P. R. China

**Keywords:** Nanoparticles, Pollution remediation

## Abstract

N-doping carbon quantum dots were successfully loaded on P25 nanoparticles (denoted as N-CDs/P25) by facile hydrothermal process, and their morphology and chemical structure were systematically studied. The carrier of N-CDs can significantly broaden the photoresponse range of the P25 to the visible region, accelerate charge transportation and separation. Application of the N-CDs/P25 material for the photocatalytic decomposition of Rhodamine B (RhB) gave improved activity relative to P25. The best degradation activity obtained at 6mL N-CDs/P25 under visible light irradiation, which shows a 13.06 fold photocatalytic activity over P25. Radical trapping control experiment and Electron Paramagnetic Resonance (EPR) measurements have been applied to explore the photodegradation dynamic and visible-light driven degradation mechanism. This work provides new insights into the fabrication of N-doping carbon quantum dots/TiO_2_ composite and is promising to open new possibilities in the application of carbon-TiO_2_ composites as the photocatalysts in the environmental protection issues.

## Introduction

TiO_2_ are the most widely explored semiconductor photocatalysts in environmental pollutants treatment and solar-energy conversion area in these latest decades for their superior photocatalytic activity, nontoxicity, long-term stability and low cost^1^. Degussa (Evonik) P25 is a biphasic titanium dioxide that owns especially high photocatalytic activity due to its special biphasic interfacial heterojunction structure^2–4^. It contains more than 80% anatase with a minor amount of rutile (15%) and a small amount of amorphous phase with approximately 20 nm particle size^5,6^. Interfaces between rutile and anatase phase facilitate charge carrier separation and retard the charge recombination between electron trapping sites within the mixed phase so as to improve photocatalytic activity^7–10^; Therefore P25 is prevailing used in laboratory for photocatalytic reaction systems. However, P25 have its own inherent weakness, for example, the large band gap limited its response wavelengths in the UV region, lower absorption volume and slower electronic mobility limited its energy conversion efficiency^11,12^. A strategy we can take is the combination of the photoactive P25 nanoparticle with visible light adsorptive conductor. Not only does this provide visible light sensitizer and electron trapping sites, extend its photoresponse wavelength to visible region, it also ensures relative fast electronic transportation to the reaction interface that is beneficial to charge separation, enhance the conversion efficiency^13,14^.

Carbon quantum dots (CDs) are zero dimensional nanocarbon material less than 10 nm in diameter, formed by crystalline graphitic sp^2^-bonded carbon atoms, which have a quantum confinement effect^15–18^. Apart from its unique electronic attributes, the quasi-spherical nanocarbon material possess several other attractive advantages, such as the large specific surface area, high stability, low toxicity, simple synthetic routes and the strong and tunable fluorescence emission properties^19–22^. Based on those novel properties, therefore, the couple of P25 and CDs is anticipated to simultaneously have high photo activity, excellent adsorptivity, good electronic mobility and good cost-efficiency, therefore enhancing the photocatalytic decomposition abilities of pollutants^23–26^. Herein we demonstrated a facile route to prepare a chemically bonded N-doping carbon quantum dots/P25 composite via hydrothermal method that is illustrated in Scheme 1. The as-prepared composites own several advantages over other catalysts: the enhanced adsorptive ability of pollutants, fast charge conductivity and separation, extended light absorption range. The factors of particle size, chemical composition, and the CDs content in N-CDs/P25 that may influence the catalytic performance were systematically discussed. The as synthesized N-CDs/P25 composites show higher photocatalytic activity in the degradation of Rhodamine B (RhB) over the commercial P25 catalysts under visible light illumination. Finally based on trapping control experiment and Electron Paramagnetic Resonance (EPR) measurements, we proposed one feasible visible-light driven degradation mechanism.

## Experimental Sections

### Synthesis of N-CDs quantum dots

Experimental procedure of N-CDs fabrication have been reported elsewhere in the literature and here cited with minor modifications^27–29^. Typically 4 mmol citric acid and 12 mmol urea were dissolved together in 20 ml distilled water, and then poured into a 100 ml Teflon lined hydrothermal reaction vessel. The reaction take place at 200 °C and kept for 8 hours. Then Cool to ambient temperature and adjusting the pH to 7. Centrifuge to get a dark yellowish N-CDs solution. The solution was put in a dialysis tube (keep molecular weight: 500 Da~3500 Da) for 4 days in the dark (changed deionized water every 8 h). Concentration to 20 mL.

### Synthesis of N-CDs/P25 composites

N-CDs/P25 was synthesized via a hydrothermal deposition process. Typically, 0.2 g P25 was put into certain volume of as-prepared N-CDs solution and added 40 mL more water, mixed and transferred to a dried Teflon-lined hydrothermal reaction vessel, heated to 200 °C and kept for 6 hours. After cooling down the mixture were then centrifuged at 8000r/min to collect the final product. The samples were named according to the added volume of N-CDs as (2 mL)N-CDs/P25, (4 mL)N-CDs/P25, (6 mL)N-CDs/P25, (8 mL)N-CDs/P25 and (10 mL)N-CDs/P25

### Photocatalytic performance evaluation

The photodegradation of Rhodamine B (RhB) were chosen as model reaction to evaluation the photocatalytic activities of the as synthesized material under visible light irradiation. Xenon lamp with a <400 nm cutoff filter was used as the photo source to mimic the nature solar irradiation. Generally, a 150 mL quartz reactor were equipped with a thermostat to keep the degradation temperature at 28 °C, in which put 10 mg/L RhB100 mL. Into the RhB solution added 50 mg catalysts. Keep the mixture in the dark for 30 min to make sure the catalysts saturated with the adsorption of RhB. Turn on the lamp to start the photocatalytic reaction and accumulate the irradiation time. To trace the dynamic concentration changes of the RhB 3 mL of the mixture each time was taken out from the reactant at regular intervals. The concentration was obtained by UV-VIS measurements.

## Results and Discussion

**Figure 1:**
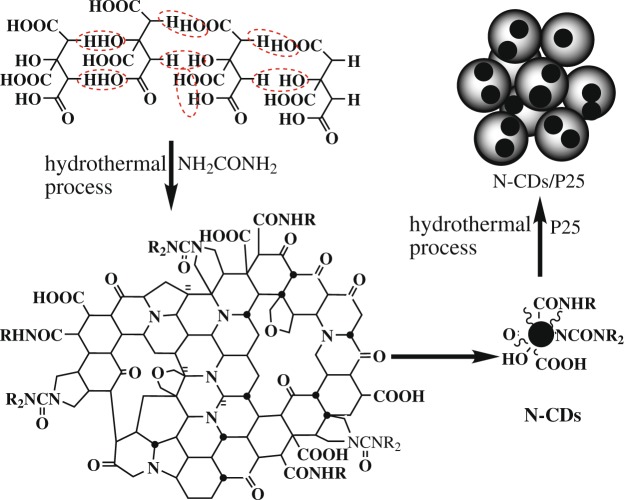
Illustration of procedures for preparation of the photocatalysts N-CDs/P25 by hydrothermal deposition.

The synthetic route for N-CDs/P25 composites is depicted in Fig. 1. Firstly citric acid was carbonized with hydrothermal process to form N-doped CDs and was purified through centrifugation and dialysis. Then mixed the N-CDs solution and P25 with hydrothermal reaction, Ti-OH on the surface of P25 dehydrates easily with the C-OH groups on the N-CDs surfaces, chemically formation of the N-CDs/P25 composites.

**Figure 2:**
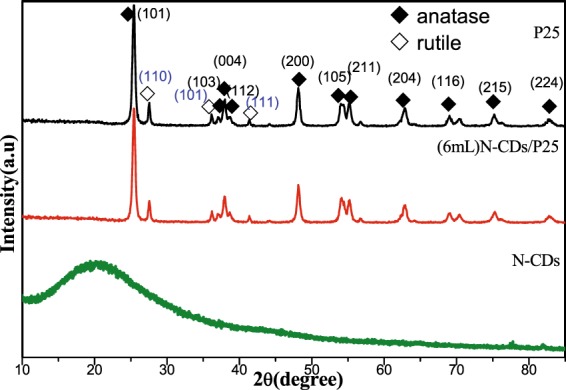
XRD spectra of N-CDs, P25 and N-CDs/P25. ◆ stands for anatase, ◇ for rutile.

XRD were carried out to study the crystallographic phase structures of the as prepared catalysts. Figure 2 shows the measured XRD measurements of N-CDs, P25 and N-CDs/P25 respectively. The bottom curve at 21 tin Fig. 2 occurs a broad peak, which is assigned as the (002) crystal plane of graphite. The measured interlamellar spacing of as-synthesized N-CDs is bigger than that of graphite. the nitrogen-containing groups introduced into the interlayer space may account for this effects.

Meanwhile the composites (6 mL)N-CDs/P25 in Fig. 2 shows that no N-CDs peak was detected; this may be attributed to the situation that the C quantum dots is greatly scattered on P25 nanoparticles. In addition the low doping content of C quantum dots is another factor that caused the disappearance of N-CDs signals in the XRD measurement.

Figure 2 also shows, the diffraction pattern for (6 mL)N-CDs/P25 and P25 are identical, matching well with the standard mixture phase of anatase and rutile-phase TiO_2_ (JCPDS No. 84-1286 and No. 88-1175), indicating the coexists of the anatase and rutile phase in the N-CDs/P25 catalysts, the incorporation of N-CDs on the surface of TiO_2_ haven’t change the crystal structure of P25 powders.

**Figure 3:**
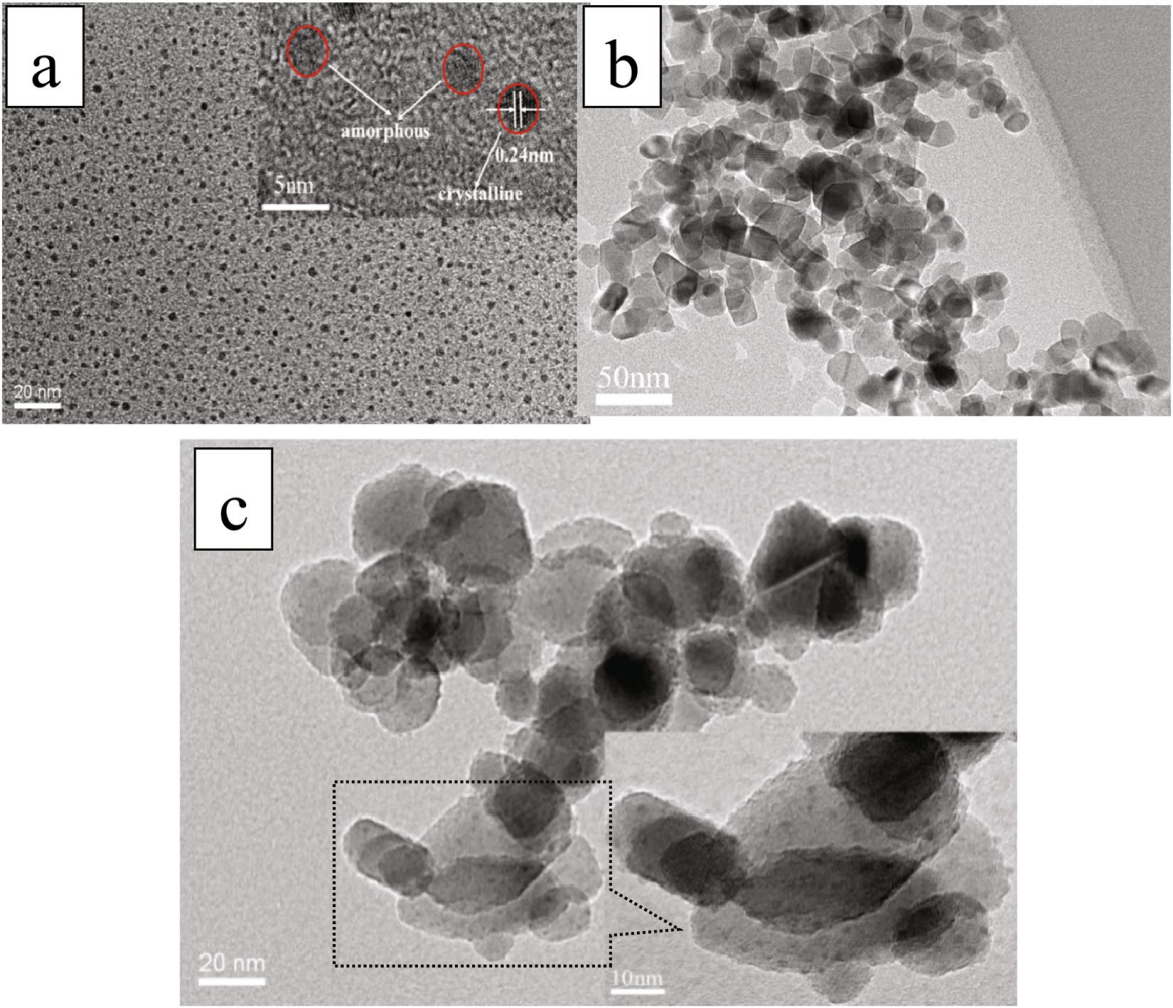
TEM measurements of (**a**) N-CDs (Inset is the high resolution image) (**b**) P25 (**c**) N-CDs/P25 (Inset is the high resolution image).

Figure 3a shows that the size of N-CDs nanoparticles is uniform and high dispersed. The particle size is approximately 3 nm in diameter. Inset of Fig. 3a is the N-CDs nanoparticles’ high resolution image, it indicates that the lattices of N-CDs is 0.242 nm. Reference assigned these lattices to the N-CDs sp^2^ clusters^30^. Figure 3b shows the TEM image of pure P25, the average diameter is about 20–30 nm. Figure 3c is the TEM measurement of the N-CDs/P25 catalysts; it demonstrates that the N-CDs are highly dispersed on P25. This uniformly distribution may result from our superiority of hydrothermal deposition procedure: crucial homogeneously filled hydro vapor pressure and the monodispersed spatial confined P25 surfaces.

**Figure 4:**
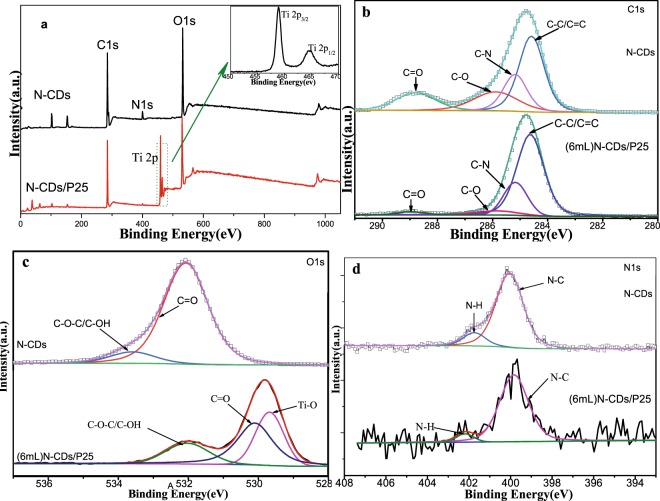
XPS characterization of N-CDs and N-CDs/P25. (**a**) full survey scan XPS pattern, (**b**) C 1 s narrow scan XPS spectra and (**c**) O 1 s narrow scan high resolution XPS spectra (**d**) N 1 s narrow scan XPS spectra.

X-ray photoelectron spectroscopy (XPS) measurement was carried out for N-CDs and N-CDs/P25 to determine the valence states of the related elements, and the results are shown in Fig. 4. Survey scan in Fig. 4a suggests that the samples of our synthesized N-CDs or N-CDs/P25 both contain Oxygen, Nitrogen and Carbon elements. XPS of N-CDs/P25 indicates the existence of Ti 2p coming from TiO_2_. Moreover, inset of Fig. 4a shows the wide scan oxidation state of the Ti in N-CDs/P25. Two distinct bands located at 459.08 eV and 465.08 eV are assigned to the Ti2p3/2 and Ti2p1/2 signals in the Ti^4+^ valence state, respectively^31^. Because of the small size of CDs (<3 nm), the XPS can reach more than the whole CDs particles, so the collected XPS spectrum origins from the whole catalysts, rather than the surface alone.

The XPS spectra and deconvoluted fitted peaks for C,O and N were shown in Fig. 4b–d, respectively. The C 1 s XPS spectrum shown in Fig. 4b was deconvoluted fitted as four peaks: The binding energy observed at 285.88 eV was attributed to C–O group, binding energy at 288.78 eV was assigned as the C=O carbonyl group, energy at 285.18 eV ascribed to C-N, the peak at 284.58 eV comes from the C–H/C=C/C–C group. The above deconvoluted fitted spectra indicate that N-CDs and N-CDs/P25 composites are rich of O element and N element. Figure 4 shows that the intensity of O signal in the C XPS spectrum decreased for N-CDs/P25 relative to N-CDs, which means that P25 have been covered by N-CDs. The XPS pattern of O 1 s shown in Fig. 4c indicates the great difference between N-CDs and N-CDs/P25. N-CDs in top panel of Fig. 4c shows, the binding energy at 533.59 eV ascribed to the C-O and -OH, deconvoluted peak at 532.07 eV were related to carbonyl group, after integration with P25, as bottom panel shows, these peaks were shifted towards lower order values. 529.68 eV for Ti-O, 530.08 eV for carbonyl group, 532.08 eV for C-O and -OH, respectively. The above comparisons in XPS spectra demonstrate the strong interaction between the TiO_2_ and C after the solvothermal process and further confirm the successful integration of N-CDs on P25. The N 1 s peaks detected at 400.08 and 401.78 eV in Fig. 4d demonstrate that N element presents primary in N-H (401.78 eV) or N-C(400.08 eV) style, respectively, either on the N-CDs or N-CDs/P25. Specifically there is no C−Ti peak detected, which means N-CDs integrated to P25 were not directly trough C–Ti bond, but takes the form of Ti-O-C.

**Figure 5:**
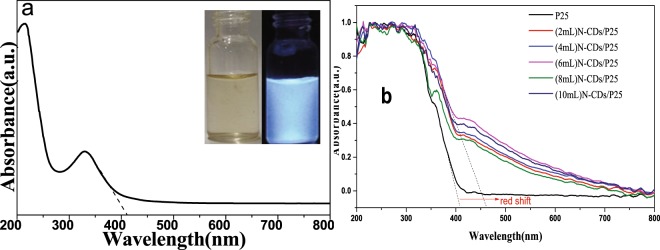
UV-vis Spectra (**a**) Absorption spectrum of N-CDs dispersion (Include the pictures of N-CDs dispersion taken under nature solar light and 365 nm light illumination respectively). (**b**) Comparison of UV-vis diffuse reflectance spectra of various as prepared N-CDs/P25 nanoparticles.

200–800 nm absorption spectrum of N-CDs solution and the pictures of N-CDs illuminated under visible light and 365 nm light are shown in Fig. 5a. π–π* transition of C=C bonds absorbs strong band center around 228 nm region wavelength and n–π* transitions of C=O and C=N bonds shows weak wide absorption ranging from 280 nm to 450 nm^29^. Any excitation light sited between this wide absorption range can resonant with the electron and excite it to the S1 state, so light wavelength from 280 nm to 450 nm can excite to produce the photoluminescent emission of the N-CDs. It is well known that N-doping carbon quantum dots-based materials have one characteristic property, that is, fluorescence spectra is dependent on the excitation wavelength^29^. The N-CDs dispersion displays yellow color by nature light irradiation but blue color by a 365 nm light illumination as is shown in Fig. 5a right panel.

Figure 5b demonstrates the UV-vis DRS spectra of P25 and N-CDs/P25 catalysts. They all show strong absorption in the UV region, while only the N-CDs/P25 have medium absorption band across the whole visible light region, among which (6 mL) N-CDs/P25 has the highest visible light absorption intensity. Obviously the absorption edge for N-CDs/P25 shows a red shift relative to P25. This phenomenon of visible region absorption band and absorption edge red shift can be explained by the fact of N-doping CDs deposition on the catalysts, decreased the light reflection.

**Figure 6:**
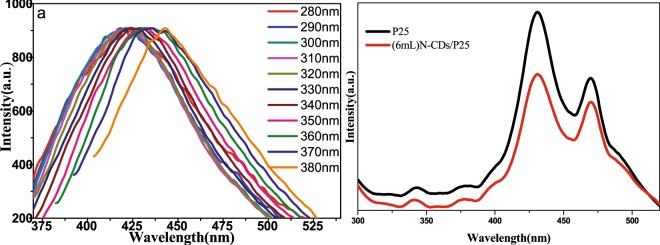
(**a**) Emission fluorescence spectra of N-CDs in water excited with series laser wavelengths, Intensity were normalized for better comparison. (**b**) Fluorescence spectra of P25 and (6 mL)N-CDs/P25.

Figure 6a shows a series of fluorescence spectra of as synthesized N doping C quantum dots pictured by different excitation wavelengths with their intensity normalized. The broad fluorescence spectra range from 350 nm to 550 nm. In addition, the emission spectra show typical excitation laser wavelength dependent property, which suggests its complicate energy band composition, either in ground state or excited band. The XPS detected carbonyl and C-N groups in N-CDs will influence the energy band structures of C-dots sp^2^ kernel and introduce additional energy stage^29^. These multiple electronic structures mix together and result in multiple electronic transition possibilities, broaden the absorption range. The probability of each transition is depends on the excitation wavelength. The excited electrons come back to their own ground state with radiative transition, producing multiple fluorescence emissions. The weights of excited resonant electrons satisfy Gaussian distribution, centered with excitation laser wavelength. The weight of each transition is dependent on the excitation laser, correspondingly the weight of each fluorescence emissions also changes with the excitation wavelength, result in the wavelengths dependent properties. It is well known that doping of the N element in C dots can reduce the band gap. Furthermore, the chemical linking of N to C will introduce additional energy level that can trap the e^−^–h^+^ pairs, thus prolong the lifetime of separate e^−^–h^+^ pairs, spare enough time for separated e^−^ conduct to other chemical reaction path. Figure 6b demonstrates that the fluorescence intensity of P25 decreases after the doting of N-CDs, it means the doting process have altered the electron radiative path and quenched the fluorescence.

**Figure 7:**
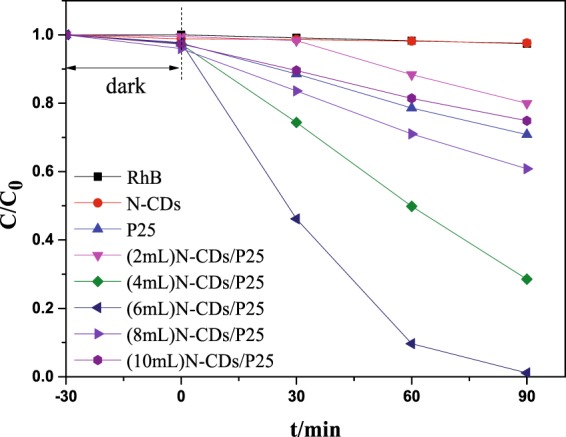
The dynamic photocatalytic degradation of RhB vs time with the visible light illumination.

**Figure 8:**
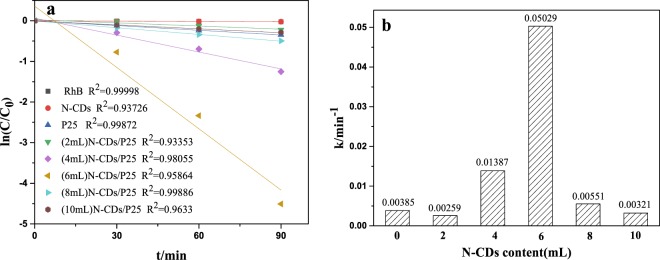
(**a**) Kinetic fitting using First order reaction function. (**b**) The obtained apparent k for as-prepared photocatalysts with different N-CDs content.

Rhodamine B(RhB) is one kind of the important dyes that has been popular applied in industrial application, which forms the pollutions to environments. In this article the degradation of RhB was chosen as the model reaction. The photocatalytic performance was compared among N-CDs**/**P25 photocatalysts, N-CDs and P25. Figure 7 shows the concentration changes of RhB with degradation time. No RhB self degradation was observed under visible light irradiation(400~780 nm). In addition there is no obvious RhB degradation either under visible light irradiation over N-CD catalysts. Approximately 30% concentration decrease was observed over P25 within 90 minutes. In contrast, the N-CDs/P25 catalysts showed higher visible-light degradation activity. Specially the RhB were completely removed by (6 mL)N-CDs/P25 within 90 minutes. It is interestingly to stress that the RhB removal activity increases with the series of N-CDs from 2 mL to 6 mL. However, further increasing the loading content will decrease the catalytic performance. The linear fitting of ln(C/C_0_) versus time (see in Fig. 8a) based on the simplified Langmuir-Hinshelwood model suggests that the RhB removal dynamics obeys a first order kinetic. The apparent rate constant (k) obtained from the linear fitting were shown in Fig. 8b. The highest k is obtained from (6 mL)N-CDs/TNS catalyst, approximately k = 5.029 × 10^−2^ min^−1^, which is 13.06 folds photocatalytic activity improvement over P25 (k = 0.00385 min^−1^). The obtained apparent rates constants suggest that there is a optimal N-CDs loading content on P25 for optimize the photocatalytic performance. The integration of N-CDs on P25 gives the photocatalysts several prominent features: improved catalysts adaptability, increased adsorption ability of pollutants, extension of light absorption edge to visible region and prolonged lifetime of photo generated electron-hole pair.

**Figure 9:**
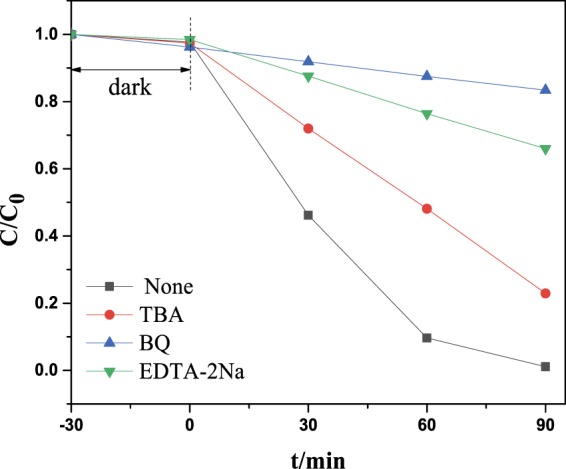
Active species capture experiment for (6 mL)N-CDs/P25 by the addition of different scavenges.

To better understanding the photodegradation mechanism, especially to know what kind of reactive mediums that triggered the light induced degradation and which one be responsible for the whole kinetic photodegradation process, another experiment should be done by introducing specific radical scavenger in reaction system before the photo reaction starts^32,33^. Generally tert-Butanol (TBA) can trap OH radicals, benzoquinone (BQ) can capture O_2_^−^ radicals, EDTA-2Na is a holes scavengers and AgNO_3_ is an electrons scavenger. Figure 9 shows that, the RhB degradation under visible light irradiation was significantly suppressed when BQ(O_2_^−^ scavenger) or EDTA-2Na(holes scavenger) was added to the reactant. In contrast, the RhB degradation was slightly suppressed when TBA was added. This indicated that the ${}^{.}{\rm{O}}_{2}^{-}$ radical·OH radical and h^+^ could be the mostly possible reactive species in the photo degradation process.

**Figure 10:**
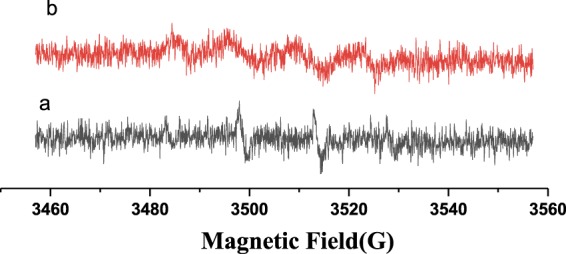
EPR spins trapping spectra for N-CDs/P25 in water (a panel) and in methanol (b panel) under visible light irradiation.

Moreover the electron paramagnetic resonance (EPR) measurements were performed to demonstrate the reactive radicals that may take charges of the degradation of RhB. Figure 10a shows the DMPO spin trapping spectra of (6 mL)N-CDs/P25 dispersed in water upon continuous laser illumination, Fig. 10b shows the compared spectra dispersed in CH_3_OH. Figure 10a indicates no characteristic signals, which suggests no DMPO-${}^{\cdot }{\rm{O}}{\rm{H}}$ adducts in water solution^34^. Figure 10b shows four signals with equal intensity. These pattern of signals are typical feature for the 5,5-dimethyl-1-pyrrolidone-2-oxyl (•DMPO-X, ie O_2_^−•^/•OOH^35^), demonstrating the formation of DMPO-${}^{\cdot }{\rm{O}}_{2}^{-}$. This experiment verifies that ${}^{\cdot }{\rm{O}}_{2}^{-}$ is the most important active redicals that created with continuous laser illumination and take charge of the photo degradation process, which is in consistency with the scavenges experiments shown in Fig. 9.

**Figure 11:**
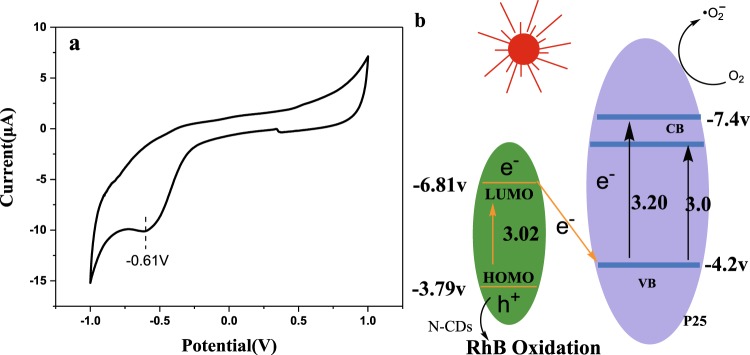
(**a**) cyclic Voltammetry curves (CVs) of N-CDs. (**b**) The proposed photo induced electron dynamic pathway of N-CDs/P25 with vis-light irradiation.

The CVs photogram of N-CDs were shown in Fig. 11a. The electrochemical behavior is reversible. From the Fig. 11a we can get the reduction potential value is −0.61 V. with the equation E_LUMO_ = −e (E_red_ + 4.4) we can get the LUMO energy is −3.79 V, which is in consistency with the reference reported^36^. Based on the UV–VIS DRS spectra shown in Fig. 5 and LUMO energy, the HOMO energy can be known E_HOMO_ = −6.81 V. Z-scheme can be drawn based on the energy data, according to the further active trapping results (Fig. 11b). The visible light induced dynamic pathway of electron can be described using Z-scheme heterojunction structures as following: the N-CDs electron in HOMO was excited to LUMO by visible light irradiation. Meanwhile, P25 can absorber the 400 nm light and the electrons are excited to the CB state due to the weak absorption “tail”. Moreover the RhB dye self-sensitization on surface of photocatalysts also lowered the band gap of p25 and favors the visible light absorption. In sum, the ground electrons on both sides could be excited by visible light to produce e^−^h^+^ separation couple. Then the produced e^−^ in LUMO orbital can annihilate the h^+^ in VB of P25, the e^−^ in CB of P25 and the h^+^ carriers in HOMO of CDs were spatially separated. This process favors the e^−^ accumulation on P25 rather radiative transition to VB. Because no h^+^ are remaining on P25, much longer lifetimes of the hot electrons on the CB could be expected, thus increase the probability to reduce the O_2_ adsorbed on the P25 and helps the photo degradation of RhB. In the meantime, the h^+^ carriers of N-CDs HOMO orbital may attack directly the dye pollutions, or react with H_2_O to form ${}^{\cdot }{\rm{O}}{\rm{H}}$. Sum up, the Z-scheme heterojunction structure favors the separation of the photo induced electron–hole pairs, with the enhanced photocatalytic activity for N-CDs/P25.

**Figure 12:**
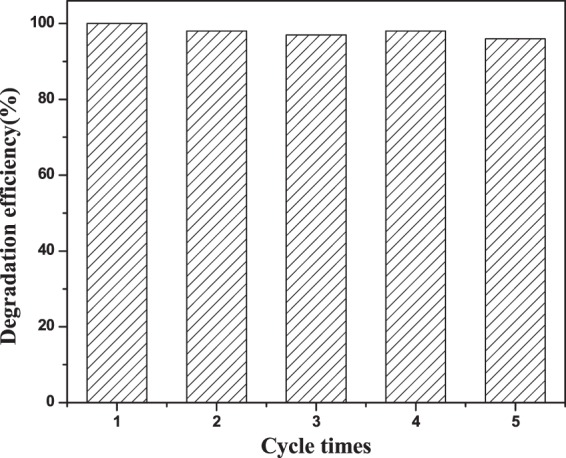
The catalysts stability measurement of (6 mL)N-CDs/P25.

The stability and recyclability of the catalysts have been carried out and the results have been shown in Fig. 12. The degradation efficiency of RhB exhibits high stability after 5 successive continuously recycles of the catalysts under identical conditions. Intrinsic biphasic structure of P25, the application of most affinity force size of the N-CDs and the strong interaction between the P25 and N-CDs heterojunction may account for this superior catalytic stability.

## Conclusion

Overall, the TiO_2_ nanoparticles loaded with N doping carbon quantum dots-N-CDs/P25 composites-were synthesis using hydrothermal method to extend their photoresponse range and improve photocatalytic stability. Our results showed that the precipitation of N-CDs on P25 could considerably enhance the visible-light photocatalytic activity of P25. The screened best catalyst is (6 mL)N-CDs/TNS, its degradation rate constant was found to be 13.06 folds of pure P25. TEM confirmed the as prepared N-CDs/P25 composites structure; the N-CDs are spherical and highly dispersed with a mean size of approximately 3 nm. UV-vis DRS spectra verified the efficient absorption edge extension from UV to visible light region. XPS measurements proved that the N-CDs have been chemically bonded to P25 by C-O-Ti. The high photocatalytic can be attributed to the synergistic effects of the Z-scheme structure, the biphasic heterojunction of P25, the extension of visible light absorption edge, and the prolonged e^−^h^+^ separation lifetime. The as-prepared N-CDs/P25 catalyst is anticipated to be applied in photocatalysis water treatment, photocatalytic synthesis, solar cells and sensors etc.
